# Combined Dietary Nitrate and Exercise Intervention in Peripheral Artery Disease: Protocol Rationale and Design

**DOI:** 10.2196/resprot.7596

**Published:** 2017-10-03

**Authors:** Mary N Woessner, Mitch D VanBruggen, Carl F Pieper, Erin K O'Reilly, William E Kraus, Jason D Allen

**Affiliations:** ^1^ Clinical Exercise Science Research Program Institute of Sport, Exercise and Active Living Victoria University Melbourne Australia; ^2^ Duke Molecular Physiology Institute Duke University Medical Center Durham, NC United States; ^3^ Duke University Medical Center Department of Biostatistics and Bioinformatics Duke University Durham, NC United States; ^4^ Office of Regulatory Affairs and Quality Duke University Medical Center Durham, NC United States

**Keywords:** nitrate, nitrite, nitric oxide, exercise, peripheral arterial disease, intermittent claudication

## Abstract

**Background:**

Peripheral artery disease (PAD) is caused by atherosclerotic occlusions in the legs. It affects approximately 8-12 million people in the United States alone, one-third of whom suffer from intermittent claudication (IC), defined as ischemic leg pain that occurs with walking and improves with rest. Patients with IC suffer a markedly impaired quality of life and a high perception of disability. Improving pain-free walking time is a primary goal of rehabilitation in this population.

**Objective:**

The nitric oxide (NO)-PAD trial is designed to compare the effects that 12 weeks of supervised exercise training, in combination with a high inorganic nitrate-content (beetroot [BR] juice) beverage or placebo (PL) beverage, has on clinical outcomes of exercise and functional capacity in two groups of PAD+IC patients: exercise training plus beetroot (EX+BR) and exercise training plus placebo (EX+PL). The primary aims of this randomized controlled, double-blind pilot study are to determine group differences following 12 weeks of EX+BR versus EX+PL in the changes for (1) exercise capacity: pain-free walking time (claudication onset time, COT), peak walk time (PWT), and maximal exercise capacity (peak oxygen uptake, VO_2peak_) during a maximal-graded cardiopulmonary exercise test (max CPX) and (2) functional capacity: 6-minute walk (6MW) distance. The secondary aims will provide mechanistic insights into the exercise outcome measures and will include (1) gastrocnemius muscle oxygenation during exercise via near-infrared spectroscopy (NIRS); (2) gastrocnemius muscle angiogenesis: capillaries per unit area and per muscle fiber, and relative fraction of type I, IIa, IIb, and IId/x fibers; and (3) vascular health/function via brachial artery flow-mediated dilation, lower-limb blood flow via plethysmography, and pulse wave velocity and reflection.

**Methods:**

A total of 30 subjects between 40 and 80 years of age with PAD who are limited by IC will undergo exercise training 3 days per week for 12 weeks (ie, 36 sessions). They will be randomized to either the EX+BR or EX+PL group where participants will consume a beverage high in inorganic nitrate (4.2 mmol) or a low-nitrate placebo, respectively, 3 hours prior to each training session.

**Results:**

Data collection from this study has been completed and is in the process of analysis and write-up. While the study is too underpowered—EX+BR, n=11; EX+PL, n=13—to determine between-group differences in the primary outcomes of COT, PWT, and 6MW, preliminary observations are promising with Cohen *d* effect sizes of medium to large.

**Conclusions:**

Exercise training is currently the most effective therapy to increase functional capacity in PAD+IC. If the addition of inorganic nitrate to an exercise regimen elicits greater benefits, it may redefine the current standard of care for PAD+IC.

**Trial Registration:**

ClinicalTrials.gov NCT01684930; https://clinicaltrials.gov/ct2/show/NCT01684930 (Archived by WebCite at http://www.webcitation.org/6raXFyEcP)

## Introduction

Peripheral artery disease (PAD) is caused by stenosis of the arteries in the lower limbs, leading to a reduction in blood flow to the legs [1]. It affects approximately 8-12 million people within the United States alone, one-third of whom suffer from intermittent claudication (IC), defined as ischemic leg pain that occurs with walking and improves with rest [2]. Patients with IC suffer a markedly impaired quality of life and a high perception of disability [3]. Consequently, improving pain-free walking time is an important clinical goal for these patients [4].

Thus far, interventions aimed at improving clinical outcomes in patients with PAD have been either surgical, pharmacological, or exercise based, with supervised exercise training identified as the current best intervention for improving functional outcomes such as peak walking time and pain-free walking time [5-7]. Exercise training has proven efficacy in improving vascular health and function for atherosclerosis-associated comorbidities such as obesity, hypertension, dyslipidemia, and type 2 diabetes mellitus [8-14]. Patients with PAD have marked impairments in the oxygen delivery and uptake pathway due to deficiencies in both vascular function—increased arterial stiffness, endothelial dysfunction, and decreased peripheral blood flow—and skeletal muscle composition and architecture—capillary density rarefaction, mitochondrial dysfunction, and a loss of oxidative (ie, slow-twitch) fibers [15-18]. As they result in a more glycolytic phenotype leading to earlier onset of fatigue and exhaustion, the skeletal muscle abnormalities have a detrimental effect on the delivery, uptake, and utilization of oxygen. While supervised exercise training can improve both the skeletal abnormalities [19] and vascular function [20] in these patients, improving the patient’s ability to acutely tolerate an exercise bout could lead to even greater efficacy in the exercise training intervention by allowing them to exercise at a greater intensity at each session. Thus, to identify the next best treatment for patients with PAD, there is a continued need to advance our current understanding of both the disease and optimal interventional therapies.

Dysfunction of the vascular endothelium is a hallmark of cardiovascular diseases, including PAD [21]. A key facet of this dysfunction is abnormal vessel reactivity, which is mediated in part by a reduction in nitric oxide (NO) production [22]. NO is diatomic free radical that plays an important role in modulating vascular tone and regulating blood flow. In healthy individuals, NO is produced endogenously by endothelial nitric oxide synthase in response to elevated shear stress at the arterial wall. Patients with cardiovascular disease, in particular those with PAD, lack the ability to endogenously increase vascular NO bioavailability, leading to significant dysfunctions within the vasculature.

Plasma nitrite (NO_2_^-^), while once considered to be a biologically inert marker of NO, has recently been identified as an alternative NO source that can be reduced to NO under low oxygen conditions, such as hypoxia or ischemia [23]. One established noninvasive mechanism for increasing plasma NO_2_^-^ is via oral consumption of inorganic nitrate (NO_3_^-^). Inorganic NO_3_^-^ is found in relatively high concentrations in green leafy vegetables and beetroot. When swallowed, inorganic NO_3_^-^ is rapidly absorbed in the small intestine and while a majority is excreted by the kidneys, up to 25% is retained and becomes concentrated in the salivary glands. When the saliva is secreted, commensal oral bacteria on the dorsal surface of the tongue reduce the NO_3_^-^ to NO_2_^-^, which is then swallowed and absorbed back into the circulation. NO_2_^-^ concentration peaks approximately 2.5-4 hours after inorganic NO_3_^-^ consumption [24,25]. The circulating NO_2_^-^ is further reduced to NO in hypoxic conditions; the NO_3_^-^ -NO_2_^-^-NO reduction pathway increases peripheral blood flow during exercise and leads to improvements in exercise tolerance.

NO_3_^-^ supplementation has shown mixed effects on exercise performance [26-30]. In healthy and athletic populations, NO_3_^-^ supplementation has decreased the oxygen cost of exercise [28,31], increased time to exhaustion [28], and improved time trial performance [26]. However, studies utilizing similar participant demographics have reported no effect of NO_3_^-^ supplementation on power output [32], time trial time [33], or submaximal exercise efficiency [34]. One proposed explanation for these mixed findings is the discovery that an individual’s level of aerobic fitness could impact the effectiveness of nitrate supplementation, with one study demonstrating reductions in the oxygen cost of exercise and improvements in time trial performance only in participants with a low-moderate level of aerobic fitness [35]. While this research into responders and nonresponders to nitrate supplementation is still in the early stages, this particular finding perhaps lends more support for nitrate supplementation’s potential efficacy in clinical populations with low aerobic capacity. Indeed, in clinical populations, most of the results have been positive: improvements in submaximal endurance, muscle contractile function, and exercise capacity in heart failure [36-38], as well as submaximal exercise endurance in chronic obstructive pulmonary disease [39]. Only a few studies showed no effect [40]. In patients with PAD, following a single acute dose of a high-nitrate-containing beetroot (BR) juice (18 mmol), which led to a five-fold increase in plasma nitrite in comparison to placebo (PL) [27], we have previously shown an 18% and 17% increase in treadmill walking claudication onset time (COT) and peak walk time (PWT), respectively. While initial results show promise, most of the studies have utilized acute dosing strategies and have been limited in both sample size and demographic diversity.

While exercise training is the current best intervention for patients with PAD, by allowing the patients to acutely train at higher intensities—due to improvements in oxygen delivery/uptake—resulting in a greater cumulative effect than exercise alone, the acute benefits of nitrate supplementation on exercise tolerance suggest a potential opportunity to further enhance the effects of exercise training [6].

The hypothesis of this study is that subjects with peripheral artery disease with intermittent claudication (PAD+IC) who undertake regular consumption of a high-inorganic nitrate (4.2 mmol) supplement in conjunction with 12 weeks of supervised exercise training (exercise training plus beetroot juice [EX+BR]) will experience a greater clinical benefit in pain-free walking time (COT, 6-minute walk [6MW]) and PWT than those undertaking exercise and a placebo beverage (exercise training plus placebo juice [EX+PL]) alone. Specifically, the primary aim of this study is to determine group differences (EX+BR vs EX+PL) in the changes in the following:

1. Exercise capacity: pain-free walking time (COT), PWT, and maximal exercise capacity/peak oxygen uptake (VO_2peak_) during a maximal-graded cardiopulmonary exercise test (max CPX).
2. Functional capacity: 6MW distance.

Secondary aims designed to provide mechanistic insight into the exercise outcomes include the following:

1. Gastrocnemius tissue oxygenation during max CPX testing via near-infrared spectroscopy (NIRS).
2. Gastrocnemius muscle angiogenesis: capillaries per unit area and per muscle fiber, and relative fraction of type I, IIa, IIb, and IId/x fibers.
3. Vascular health/function via brachial artery flow-mediated dilation, lower-limb blood flow via plethysmography, and pulse wave velocity (PWV) and reflection.

## Methods

### Study Design

**Figure 1:**
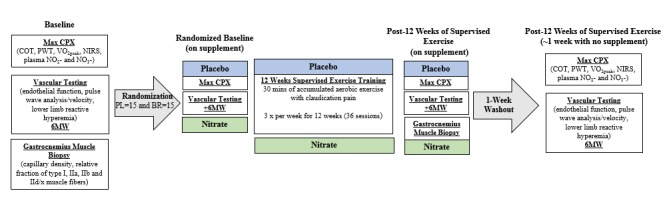
Study design with visit timeline and corresponding assessments. 6MW: 6-minute walk; BR: beetroot juice; COT: claudication onset time; max CPX: maximal-graded cardiopulmonary exercise test; NIRS: near-infrared spectroscopy; PL: placebo juice; PWT: peak walk time; VO2peak: peak oxygen uptake.

The NO-PAD trial is a pilot, randomized, double-blind, per-protocol design with four different assessment time points (see Figure 1). This study is registered with ClinicalTrials.gov (NCT01785524).

The first round of assessment will act as a baseline with no exercise intervention or supplementation. Following this, all participants will be randomized to groups consuming either a 70 mL (4.2 mmol nitrate) beetroot juice (Beet It, James White Drinks Ltd, Ipswich, UK) or an identical nitrate-depleted placebo. Participants will then repeat the baseline assessments, except for the muscle biopsy, but will consume their assigned beverage 3 hours prior to each visit.

Participants will continue consuming their beverage 3 hours prior to each of their 36 exercise training sessions. Periodically, subjects will be selected for unannounced blood draws at training visits to check plasma nitrate and nitrite concentrations. Post-exercise training, participants will complete the same assessments as the baseline visit while still acutely consuming their beverage before visits. Both groups will then stop consuming the beverage for at least 1 week prior to the final visit. The half-life of nitrate is 5-8 hours and as the supplementation protocol is three acute doses per week, a 1-week washout period should be sufficient to minimize any possible residual effect of the nitrate [41]. To maximize internal validity, study personnel, time of day, equipment, and order of testing will be consistent for each of the assessment time points.

### Recruitment Strategies and Eligibility

We aim to recruit a total of 30 participants—equal numbers of men and women—between the ages of 40 and 80 years with diagnosed PAD from the clinics and community at Duke University Medical Center. We anticipate a dropout rate of approximately 15%-20% and, therefore, 24 patients out of the 30 (EX+BR, n=12; EX+PL, n=12) are expected to complete the study. This study will have continuous rolling enrollment until the recruitment goals are met. Subjects will be recruited primarily through collaboration with Duke University Medical Center cardiovascular physicians and vascular surgeons. Clinic medical records will be reviewed to prescreen patients for inclusion and exclusion criteria (see Textbox 1).

**Inclusion and exclusion criteria for study screening visit.**

**Inclusion criteria:**

1. Aged between 40 and 80 years.

2. Diagnosed peripheral artery disease (ankle-brachial index <0.90) with intermittent claudication.

3. No major changes in medications for at least 3 months.

**Exclusion criteria:**

1. Foot ulcers, advanced neuropathy, gangrene, or other musculoskeletal condition that could limit exercise performance.

2. Type 1 diabetes or glycated hemoglobin >8.5%.

3. A major cardiovascular event within the previous 6 weeks or a planned hospitalization within the next 2 months.

4. Any cardiovascular condition that impacts safety of completing a cardiopulmonary exercise test, including history of significant left main or three-vessel coronary artery disease (>70% stenosis), recent myocardial infarction (6 weeks), chest pain during cardiopulmonary exercise test, or >2 mm ST depression during exercise; foot ulcers/advanced neuropathy or other musculoskeletal condition that could limit exercise performance.

5. Allergy to beets or proton pump inhibitors.

6. Refusal or inability to abstain from the use of proton pump inhibitors for 24 hours prior to testing.

Records of those criteria will be noted and the appropriate medical doctor will introduce the study to the patient. Patients who consent to being contacted by the research team will undergo a telephone screening questionnaire to determine if the study eligibility criteria are met. Eligible and interested patients will then attend an orientation visit and a consent meeting. During this initial visit, the study details will be explained verbally and the individuals will have the opportunity to read over the approved informed consent document and ask any questions they may have about the trial. The patients can then choose to sign the informed consent form or decline participation. Following the consent visit, enrolled subjects will return on a separate day to undergo a physical examination and a review of their medical history and medications by the study physician. Participants will then be scheduled for their baseline assessment visits. Details of the testing occurring at each time point is outlined in Figure 1 and is described in detail in the Testing Methodology section.

### Supplementation

Following initial screening and baseline testing, subjects will be randomized to consume either a high-nitrate-containing beetroot beverage or a low-nitrate placebo throughout the duration of the exercise training. Randomization should ensure the groups are initially equal within either the EX+BR or EX+PL intervention. Both groups will be instructed to consume one 70 mL bottle of beetroot juice (containing 4.2 mmol inorganic nitrate) 2.5-3 hours prior to every testing visit and training session between time point 2 and time point 3. The beetroot juice bottles will be identical in appearance and taste. The bottles will be color coded by a third party to ensure that the research staff remain blinded while providing the correct supplement to each participant.

Scheduled but unannounced blood draws will be performed throughout the study to confirm participants are conforming to their appropriate beverage allocations.

### Exercise Intervention

#### Overall Program and Attendance Requirements

All subjects will attend supervised exercise training sessions three times per week for a total of 12 weeks at the Duke Health and Fitness Center at the Duke University Medical Center for Living. Each session is structured to last approximately 45-60 minutes, including taking vital signs—heart rate and blood pressure (BP)—before and after the training session. To meet minimum exercise session attendance requirements, participants will need to complete 34 of the 36 exercise training sessions and should not miss more than 1 week of training (ie, 3 sessions) consecutively. Additional sessions will be added if necessary to ensure that an adequate exercise stimulus is given to each subject so that any decreases in fitness or function during unintended breaks are mitigated. The study will be analyzed under per-protocol criteria. Subjects who withdraw from the study prior to completion will be excluded from the final analysis. However, attrition rates will be reported and documented.

Participants will not be excluded due to current exercise habits, but all participants will be instructed to maintain their normal activity levels throughout the exercise intervention.

#### Training Details

The exercise session will consist of approximately 5 minutes of warm-up, 30 minutes of accumulated exercise (not including rest periods), and cooldown (if necessary). The walking treadmill exercise prescription is individualized to each participant’s exercise capacity during baseline testing and their rate of progression. The protocol requires participants to accumulate a total of 30 minutes of walking with claudication pain. Neither rest time nor time spent exercising before pain onset will count toward the 30-minute goal. The patient will be encouraged to continue walking as far as they can, but will self-select when they require a rest break. Exercise and rest periods are repeated during each session until the minimum total of 30 minutes of walking time is reached each session. When a patient can walk 8-10 minutes at the initial workload, the grade will be increased by 0.5%, or the speed increased by 0.1 mph as tolerated, to ensure workload progression.

Subjects will be supervised by a trained exercise physiologist and will have continuous telemetry heart rate monitoring using the Polar heart rate monitor (Polar Electro). Fatigue will be assessed through rating of perceived exertion via the Borg 6-20 scale; blood pressure will be checked both before and after exercise as well as periodically during each exercise.

Each exercise training session will be documented with preexercise and postexercise vital signs, time of day beverage was consumed, total exercise time, and workload (ie, treadmill speed and grade) recorded. Body weight and dietary intake will be assessed on a weekly basis. All research team members working with the subjects will be blinded to the treatment intervention and every attempt will be made keep encouragement and workload adjustment standardized between subjects.

### Testing Methodology

#### Overview

The subjects and research staff conducting the testing will be unaware of group assignments. The primary investigator will know to which treatment group the subjects are randomized, since changes in plasma nitrate and nitrite will be used to determine beverage volumes and tolerance. Because of the potential for bias, however, the primary investigator will not be directly involved in collecting or analyzing any of the exercise performance or training data.

For all testing visits, participants will be advised to avoid exercise and consuming alcohol for 24 hours prior to the examination day. They will also be asked to avoid tobacco and caffeine for 3 hours prior to any testing or exercise visit.

#### Maximal Cardiopulmonary Exercise Test

##### Overview

The maximal cardiopulmonary exercise test (CPX) with a 12-lead electrocardiogram and expired gas analysis will be conducted using a modified Gardner protocol. This protocol starts at 2.0 mph and 0.0% grade and increases by 2.0% grade every 2 minutes at each stage [42].

Prior to commencing the protocol, participants will be familiarized with the Borg rating of perceived exertion scale as well as the claudication pain scale. Resting measures of gastrocnemius tissue oxygenation, via NIRS, and oxygen consumption, via a metabolic cart, will be recorded.

Throughout the test, heart rate, blood pressure, NIRS, and rating of perceived exertion will be monitored. Assuming there are no adverse events, participants will walk on the treadmill until volitional exhaustion. These participants will likely be limited by leg claudication pain and, therefore, we expect to attain a VO_2peak_, rather than a maximum.

##### Measures of Pain-Free Walking Time and Maximal Exercise Capacity

The measure of pain-free walking time will be recorded as the total time walked prior to the onset of claudication pain. During the max CPX, participants will be instructed to inform a research team member when they first feel pain in their leg during exercise—this will be recorded as COT. PWT will be recorded as the total time (in seconds) that the participant walked on the treadmill. VO_2peak_ will be the average of the last 30 seconds of exercise.

##### Nitric Oxide Bioavailability Measurement (Plasma Nitrite Changes)

Prior to beginning the max CPX, a 20-gauge intravenous catheter will be placed in an antecubital vein. Approximately 5 mL of blood will be taken prior to the max CPX testing (Pre) and at 10 minutes following exercise termination (Post). Samples will then be separated into five 1 mL Eppendorf tubes containing 5 µL heparin (1-1000 U/mL) and centrifuged at 5000 g for 3-4 minutes [26]. Afterward, the plasma will be removed into five separate tubes, snap-frozen in liquid nitrogen, and stored at -80°C until analysis.

All NO metabolite concentrations will be measured within 30 minutes of defrosting by chemiluminescence using the NOA 280 nitric oxide analyzer (Sievers Instruments) as per the manufacturer’s instructions. The reductant used for nitrite analysis will be potassium iodide in acetic acid, which has the reduction potential to convert nitrite to NO; this reductant is insufficient to reduce any higher oxides of nitrogen such as nitrate and, thus, is relatively specific for nitrite. To obtain concentrations of total plasma nitrogen oxides, we will use the same apparatus with a stronger reductant, vanadium chloride in 1 M HCl, at 94°C. This reduces the sum of all nitrogen oxides with an oxidation state of +2 or higher, which is predominantly nitrate (µM), but also includes both nitrite (nM) and nitrosothiols (nM).

##### Tissue Oxygenation Measurement (Near-Infrared Spectroscopy)

NIRS has been shown to be a reproducible and reliable method to determine tissue oxygen stores in human skeletal muscle tissue [38]. The NIRS PortaMon device (Artinis Medical Systems) is a noninvasive portable tool for measuring tissue oxygenation. It works by emitting near-infrared light wavelengths of 760 nm and 850 nm that correspond to the absorption spectra of hemoglobin (Hb) and deoxygenated Hb, respectively. Depending on the oxygenation status of the tissues (ie, how much oxygenated vs deoxygenated Hb is present), the resultant spectra will change and the light fraction will be captured by a detector on the device itself [39]. This technique provides continuous snapshots of the muscle tissue oxygenation as well as percent oxygen saturation.

The tissue oxygenation will be assessed utilizing the NIRS device to obtain an index of fractional oxygen extraction—[deoxy(Hb + myoglobin)]—and percent oxygen saturation at rest at the beginning of the exercise test and continuously monitored throughout the max CPX and recovery period [26].

#### Vascular Function

##### Overview

For the vascular testing visit, subjects will be instructed to withhold all medications and consume no food for 8 hours prior to the exam. All vascular testing will be performed between 8 am and 11 am in a temperature-controlled room following 10-15 minutes of quiet relaxation.

##### Endothelial Function (Brachial Artery Flow-Mediated Dilation)

Endothelial dysfunction is a precursor to the development of atherosclerosis and an independent predictor of cardiovascular events and clinical outcomes [43]. Brachial artery flow-mediated dilation is the most commonly used noninvasive measure for the assessment of endothelial function [44]. This technique relies on the reactive hyperemic response where there is an increase in arterial blood flow following a period of ischemia (ie, occlusion). The dilation of the artery—typically the brachial artery is imaged—represents, in part, the NO-mediated arterial response to sheer stress [43,45]. All imaging will be performed at the brachial artery of the left arm with the subject in a supine position, with the forearm extended and slightly supinated. All image captures will be r-wave triggered. Two 10-second video-captures of the brachial artery will be obtained at baseline (resting), and then 2 minutes of continuous imaging will be recorded following 5 minutes of distal forearm occlusion (reactive hyperemia). Absolute and relative changes in brachial artery diameter will be calculated as follows:

Absolute and relative changes in brachial artery diameter = (peak posthyperemia diastolic diameter − baseline diastolic diameter)/baseline diastolic diameter × 100 (1)

##### Vascular Stiffness

Central blood pressures—the pressures by which the internal organs are perfused—have been shown to be strongly related to clinical outcomes [46]. A specially designed brachial cuff will be utilized to capture brachial systolic and diastolic pressure as well as the pulse waveform. The SphygmoCor software version 8.0 (AtCor Medical) will then use a validated generalized transfer function to derive central diastolic and systolic blood pressure, mean arterial pressure and pressure product along with the pressure augmentation (ΔP) due to wave reflection, and the pressure augmentation index (AIx):

AIx = ΔP/pulse pressure × 100 (2)

To capture PWV, sequential measurements of arterial pressure waves will be taken at the carotid artery, using applanation tonometry, and femoral artery, using a specialized thigh cuff. The surface distances from the sternal notch to the carotid and femoral sites will be measured and input into the software for calculation of the PWV. Pressure wave transit times to each site will be measured using the foot-of-the-wave method:

Distance of pulse wave (DPW) = sternal notch distance to femoral artery − carotid artery distance to sternal notch (3)

Carotid − femoral PWV = DPW/transit time(s) (4)

##### Ankle-Brachial Index

The ankle-brachial index (ABI) is the ratio of blood pressure in the feet in comparison to the arms and is linked with mortality and morbidity rates [47,48]. Measurements will be obtained on both the left and right side of each subject. A 5-7 MHz handheld Doppler probe coupled with a BP cuff—positioned proximal to the probe—will be used to detect blood flow through the arteries. On each arm, the brachial artery will be occluded at the biceps using an appropriately sized BP cuff, while the Doppler probe will be placed over the brachial artery to detect blood flow. An additional cuff will be placed approximately 3 cm above the medial malleolus in order to occlude the anterior tibial (AT) artery. The AT artery bifurcates in the foot into the dorsalis pedis (DP) and the posterior tibialis (PT) arteries, so both the DP and PT arteries will be measured separately via Doppler after occlusion of the AT artery using the BP cuff. The average of the least two separate measurements will be taken at each artery. The ABI value will be calculated by dividing the higher average DP or PT value from each side by the highest average radial artery value obtained from either side [27].

##### Lower-Limb Blood Flow

Following the ABI procedure, the participant will be kept supine throughout the plethysmography measurements. Lower-limb blood flow will be assessed both at rest and following reactive hyperemia via the use of a Hokanson A16 mercury-in-silastic strain gauge plethysmograph (Hokanson Inc). Initial setup requires the patient to lie supine, with the legs elevated slightly above the level of the heart to facilitate venous emptying. Two BP cuffs will be placed on the upper thigh of the leg to be assessed: one will be used for arterial occlusion and one for venous occlusion. A mercury-in-silastic strain gauge will be placed around the widest part of the calf to allow for the indirect measurement of blood flow via changes in lower-limb diameter. Resting measures will be acquired by inflating and deflating the venous occlusion thigh cuffs to approximately 50 mmHg (ie, just above venous pressure) every 10 seconds for five cycles. During inflation, the mercury-in-silastic strain gauge will be placed under stress, which will then be graphically represented as a pressure response curve on the Hokanson A16 machine. The average of three resting measures will be used as the baseline arterial inflow measure. Following 5 minutes of rest, the thigh cuff will be inflated to 30 mmHg above systolic pressure to induce arterial occlusion for 5 minutes. Upon release of the occlusion cuff, the same procedure used for resting measures will be initiated whereby the venous occlusion cuff will be inflated and deflated to create a pressure response curve for blood flow following reactive hyperemia [17].

#### Gastrocnemius Muscle Biopsy

Prior to muscle biopsies, all subjects will be asked to refrain from anticoagulant medications. Samples from the gastrocnemius muscle will be obtained using the Bergstrom percutaneous needle biopsy technique. Biopsy sites will be anesthetized with a 2% Xylocaine solution and a 0.5 cm incision will be made through the skin and fascia. Separate samples will be taken with approximately five passes of the needle into the muscle. All samples will then be prepared immediately by weighing and then dividing the samples for later analysis. A tissue sample of approximately 100 mg is consistently obtained with a triple pass from a single insertion; we have demonstrated that this amount of tissue is sufficient for analyzing muscle fiber composition and angiogenesis. Visible blood and connective tissue will be removed and the specimens divided longitudinally. Portions for RNA (40 mg) analyses will be frozen in liquid nitrogen and stored at -70°C. Another portion to be used for histochemical analysis (~30 mg) will be oriented such that the fibers run longitudinally, mounted on cork-embedding medium (OCT compound), and frozen in isopentane cooled with liquid nitrogen.

For the analysis of the muscle tissue, after thawing the tissue, measurements will be taken for markers of angiogenesis including the following: capillaries per unit area and per muscle fiber, endothelial cells with surrounding pericytes, and relative fraction of type I, IIa, IIb, and IId/x fibers. Additionally, oxidative capacity of the fibers will be quantified via mitochondrial volume with citrate synthase activity.

### Regulatory Issues

Informed consent has been obtained from each patient and the study has been approved by the Duke University School of Medicine Institutional Review Board. The study is being run under an Investigational New Drug application filed with the US Food and Drug Administration.

### Statistical Analysis Plan

This is a repeated-measures design, with the purpose of assessing change over time for the two intervention groups. Since this is a pilot study, the overarching goal of the analyses is to derive effect sizes (ie, effectiveness) for this intervention for a future larger, confirmatory study. We will conduct the analyses chronologically in three phases: (1) descriptive analyses of dependent and independent variables; (2) bivariate analyses of the association between group membership and the outcome measures; and (3) controlled multivariable analyses, which assess the association between groups and the outcomes controlling for important covariates. Randomization should ensure the groups are initially equal; however, we will control for important baseline variables, including the two outcomes of interest. We will not control for any variables observed postrandomization [46]. Our general analytic strategy will be to employ these models to assess differences between groups and, most importantly, assess the differences in change over time between the groups. Our general baseline model will have the following form:

Y
_it_ = β
_0_ + β
_1_ (Group) + β
_2_ (Time
_t_) + β
_3_ (Group × Time
_t_) + β
_4_ (Covariates
_it_) + ε
_it_ (5)

where Y_it_ is the change in the outcome over the time points, t is an indicator of change period (t=2,4) depending on the wave of measurement, and i is an indicator of the individual (i=1,24). β_1_ is an indicator variable indicating group membership. β_2_ is an indicator variable indicating the time of testing: baseline, baseline on supplement, postintervention on supplement, and postintervention off supplement. β_3_ is a Group × Time_t_ interaction. As listed, β_4_ is a vector of regression weights linking the design matrix of covariates—time varying and time invariant—to the outcome of interest. We will center Time_t_=0 at the final 12-week time point, which will allow us to test if there are differences at the end of the trial, β_1_=0. Follow-up analyses will assess the impact of Group × Time_t_ interaction and the functional form of the change over time. Inclusion of covariates into the model, including baseline measures, may add precision to the estimates and allow us to assess the generalizability of the effects across the covariates. The list of covariates (eg, age, gender, health events, comorbidity, and medications) will be developed prior to any analysis. This is a small sample (n=24); hence, the number of covariates we can utilize in any regression is minimal: ≈10 cases per variable [47]. We will assess the impact of each potential covariate individually without respect to group and, if significant (*P*=.10), it will be retained in models with Group, Time_t_, and Group × Time_t_ (see above).

This is a pilot study and we do not expect to declare statistical significance for any of the variables assessed under this analytic structure. However, if the target 12 subjects per group is attained—EX+BR and EX+PL; 24 total subjects—and if a power of 80% with alpha set to .05 (two-tailed) is used, then we will be able to detect a 0.826 standardized difference in the difference in the change labeled as “large” in the statistical power literature [49]. We will not control for the type I error risk inherent in testing multiple outcomes. Rather, should any outcome be declared significant, the reader will be alerted to the number of tests performed and the risk in drawing definitive conclusions with multiple outcomes and a small sample size.

As noted previously, the results from this pilot study will be used to power a subsequent, larger, adequately powered confirmatory clinical trial of the impact of beetroot juice on the outcomes listed above. The results, along with theory and clinical significance, will guide the choice of the primary outcome(s) for that trial, with appropriate control for type I error. While the results of this pilot study will prove useful in the calculation of power, we are aware of the many risks of “playing the winner” in choosing to power a subsequent trial from pilot study results only [50,51].

## Results

Data collection from this study has been completed and is in the process of analysis and write-up. While the study is too underpowered—EX+BR, n=11; EX+PL, n=13—to determine between-group differences in the primary outcomes of COT, PWT, and 6MW, preliminary observations are promising with Cohen *d* effect sizes of medium to large.

## Discussion

### Principal Findings

Improving pain-free walking and exercise capacity are key goals for the treatment and rehabilitation of patients with peripheral arterial disease [1]. This study aims to determine if the combined intervention of the current best treatment for PAD (ie, supervised exercise training) in conjunction with inorganic nitrate supplementation using beetroot juice can increase exercise tolerance more than exercise training alone (ie, placebo supplement).

The physiological basis for this trial relies on the fact that supplementation with inorganic nitrate will lead to increased plasma nitrite that can be transported throughout the circulation and is reduced to NO in tissue with a low partial pressure of oxygen. This hypoxic tissue-targeting effect may be particularly pertinent for patients with PAD+IC experiencing tissue ischemia during exercise participation. While it is well established that consuming inorganic nitrate increases circulating levels of plasma nitrite [28,52], research on the corresponding effect on key clinical outcomes such as exercise performance in clinical patients, while promising, is still limited [27,38,39].

There are three mechanisms that lend support to nitrate supplementations’ clinical utility in improving exercise performance in patients with PAD. First, acute supplementation data suggest that increasing plasma nitrite via beetroot juice improves ischemic tissue perfusion leading to increases in pain-free walking time and aerobic function [27]. Second, increased exercise time or intensity will facilitate greater training responses and tissue adaptations. Third, increased bioavailability of NO could lead to a greater tissue angiogenic response. These mechanisms are complementary to each other and may result in a greater overall exercise tolerance

### Study Limitations

The small sample size and the focus on patients with PAD+IC only limit the ability to broadly apply the results of the study to the larger PAD population and to generalize these results to subsequent studies. This study also uses a per-protocol design with the intent that data may provide the physiological proof of concept for the intervention and allow us to derive variance data and effect sizes within and between groups to inform future studies, which will utilize an intent-to-treat criterion in the analytic structure.

### Potential Impact

This study has been designed to investigate a new therapeutic approach for the treatment of PAD+IC. If successful, the results could influence current medical practice for these patients, while also providing mechanistic insights into potential physiologic targets for other interventions. Research clearly identifies supervised exercise training as being one of the most effective interventions for improving exercise tolerance in patients with PAD [1,53,54]. We hypothesize the combination of supplementation with exercise will create greater adaptations than the sum of the individual exposures alone. If true, the findings would lead to a paradigm shift in the treatment recommendations for patients with PAD.

## Abbreviations

6MW: 6-minute walk
ABI: ankle-brachial index
AIx: augmentation index
AT: anterior tibial
BP: blood pressure
BR: beetroot
COT: claudication onset time
CPX: cardiopulmonary exercise test
ΔP: pressure augmentation
DP: dorsalis pedis
DPW: distance of pulse wave
EX+BR: exercise training plus beetroot juice
EX+PL: exercise training plus placebo juice
Hb: hemoglobin
IC: intermittent claudication
max CPX: maximal-graded cardiopulmonary exercise test
NIRS: near-infrared spectroscopy
NO: nitric oxide
NO2-: nitrite
NO3-: nitrate
PAD: peripheral artery disease
PAD+IC: peripheral artery disease with intermittent claudication
PL: placebo
PT: posterior tibialis
PWT: peak walk time
PWV: pulse wave velocity
VO _2peak_: peak oxygen uptake

## Appendix

Multimedia Appendix 1 NIH Clinical and Integrative Cardiovascular Sciences Study Section: Peer review report.
