# Supplementary material for: Combined Dietary Nitrate and Exercise Intervention in Peripheral Artery Disease: Protocol Rationale and Design
Source: JMIR Res Protoc. 2017 Oct 3;6(10):e139. doi: 10.2196/resprot.7596 (PMC5645641; doi:10.2196/resprot.7596)
Supplement: Multimedia Appendix 1 [file resprot_v6i10e139_app1.pdf]

SUMMARY STATEMENT
(Privileged Communication)

PROGRAM CONTACT:
Diane Reid
301-402-3824
reidd@nhlbi.nih.gov

Release Date: **07/05/2011**

Application Number: **1 R21 HL111972-01**

Principal Investigator

ALLEN, JASON DAVID PHD

Applicant Organization: DUKE UNIVERSITY

Review Group: **CICS**
Clinical and Integrative Cardiovascular Sciences Study Section

Meeting Date: **06/16/2011**
Council: **OCT 2011**
Requested Start: **12/01/2011**

RFA/PA: **PA10-069**
PCC: **HHVP N**
Dual PCC: **KCK DUAL**
Dual IC(s): **DK, AG**

Project Title: **Increased Plasma Nitrite, Tissue Oxygenation and Functional Changes in PAD**

SRG Action: **Impact/Priority Score: 20 Percentile: 8 +**
Human Subjects: **30-Human subjects involved - Certified, no SRG concerns**
Animal Subjects: **10-No live vertebrate animals involved for competing appl.**
Gender: **1A-Both genders, scientifically acceptable**
Minority: **1A-Minorities and non-minorities, scientifically acceptable**
Children: **1A-Both Children and Adults, scientifically acceptable**
Clinical Research - not NIH-defined Phase III Trial

Project Year	Direct Costs Requested	Estimated Total Cost
1	150,000	242,978
2	125,000	202,482
TOTAL	275,000	445,460

ADMINISTRATIVE BUDGET NOTE: The budget shown is the requested budget and has not been adjusted to reflect any recommendations made by reviewers. If an award is planned, the costs will be calculated by Institute grants management staff based on the recommendations outlined below in the COMMITTEE BUDGET RECOMMENDATIONS section.

1R21HL111972-01 Allen, Jason

RESUME AND SUMMARY OF DISCUSSION: This R21 application proposes a novel treatment with high nitrite diet as a treatment option for patients with intermittent claudication. The applicant, Dr. Jason Allen, is a sufficiently experienced investigator who has assembled a highly qualified investigative team. Institutional research support is excellent. Although not required for an R21 application, the applicant provides good preliminary data supporting the proposed work. The review panel noted that this application represents “innovative treatment potential”. During discussion, it was noted that skeletal muscle involvement, per se, in the overall process may be questionable. Nevertheless, the review panel agreed that this is an innovative, strong proposal addressing an important clinical topic. Outstanding enthusiasm was voiced.

DESCRIPTION (provided by applicant): Peripheral artery disease (PAD) is caused by atherosclerotic occlusions in the legs. It affects 5% of the US population over 50 yrs, one third of which suffer from intermittent claudication (IC), defined as ischemic leg pain that occurs with walking and improves with rest. An intervention that could (A) acutely improve oxygenation to areas of ischemia and (B) chronically increase vessel growth to these ischemic areas would allow for greater exercise tolerance and compliance and facilitate greater improvements in function and quality of life. Plasma nitrite was once considered a biologically inert byproduct and marker of endothelial NO production. Recently, several studies have demonstrated an endocrine role for NO equivalents, including nitrite. These equivalents may be transported in the blood to peripheral tissue beds, where under hypoxic conditions they can be converted to NO and increase blood flow and O2 delivery. This may be pertinent in an arterial occlusive disease (PAD), especially during exercise when tissue ischemia is the limiting factor. The hypothesis of this proposal is that in subjects with PAD and IC, regular consumption of a high nitrate supplement which raises plasma nitrite, in conjunction with 8 weeks of supervised exercise training at the limb ischemic threshold (SET) will produce a greater clinical benefit (increases in COT and PWT) than placebo plus supervised exercise at the limb ischemic threshold (PET). In order to adequately develop, power and execute a larger study, the following specific aims will explore our hypothesis using 24 individuals (12 per group) with PAD and IC: Specific Aim 1a: To determine the segment of PAD patients that can achieve the desired level of plasma nitrite (>500nM at 180min post beverage consumption) at a tolerable volume for an 8 week study. Specific Aim 1b: In these subjects, determine differences in COT, PWT and VO2peak during a graded exercise test, between randomization to 8 weeks of SET or PET. Specific Aim 2: To determine differences in (a) tissue oxygenation (by NIRS) and (b) NO-derived species (plasma and RBC nitrite, nitrate, nitrosothiol), cGMP, oxidative and nitrosative stress (plasma nitrotyrosine and F2-isoprostane) during treadmill testing following SET or PET Specific Aim 3: To determine differences in gastrocenemius muscle (via biopsy) (a) angiogenesis and arteriogenesis (capillary density with surrounding pericytes, proliferating cell nuclear antigen, and apoptosis) and oxidative capacity (fiber type composition, citrate synthase activity), and (b) endothelial function (brachial artery FMD) after SET or PET.

PUBLIC HEALTH RELEVANCE: Peripheral artery disease (PAD) is a form of cardiovascular disease (CVD) caused by atherosclerotic occlusions in the legs. It affects approximately 5% of the US population over 50 yrs of age, one third of which suffer from intermittent claudication (IC), defined as ischemic leg pain that occurs with walking and improves with rest. We propose by supplementing plasma nitrite via a beverage we can (A) acutely improve oxygenation to areas of ischemia and (B) chronically increase vessel growth to these ischemic areas, would allow for greater acute exercise tolerance, ease the burden of exercise compliance and facilitate greater improvements in function and quality of life

CRITIQUE 1:

Significance: 2
Investigator(s): 2
Innovation: 2
Approach: 2
Environment: 1

Overall Impact: There are currently very few treatment options for patients with intermittent claudication. The applicants propose a novel treatment with a high nitrite diet. They have preliminary data that support this concept, and a strong research team to address this topic in a multifaceted way. This has significant potential public health benefit if functional improvements are noted.

1. Significance:

Strengths

- Intermittent claudication is an increasingly common disorder that will continue to increase in prevalence in an aging population. There are currently very few medical therapies for intermittent claudication. Given the inevitable increase in prevalence, an effective medical therapy would be a significant advancement.
- If successful, this small trial would have a very good chance of leading to a broader trial to more thoroughly address the effects of a high nitrite diet on claudication.

Weaknesses

- Other dietary factors have been addressed in the past, such as folate and homocysteine levels, with respect to their effect on PAD. While they have been shown to correlate with PAD disease severity, dietary supplements have not been shown to influence the course of the disease. Thus, it is difficult to say whether additional attempts at dietary modification will substantially change the course of disease.

2. Investigator(s):

Strengths

- Investigator is young but with extensive background in this field of research
- Co-investigators and research team provide the expertise needed to perform the research. The skills of the various investigators seem to complement each other well.

Weaknesses

- No specific weaknesses. Seems to be a very well-rounded team.

3. Innovation:

Strengths

- The proposed pharmacologic intervention utilizing beet juice extract is a novel idea in the treatment of peripheral vascular disease. The use of a low nitrite, identical tasting placebo was an essential component that the investigators seem to have very successfully addressed.

Weaknesses

- Peripheral vascular disease is a multi-factorial disorder with a number of factors influencing its course, including smoking, diabetes, hyperlipidemia, etc. There is evidence that the symptoms are not solely related to oxygen delivery, and the relationship between tissue oxygenation and physical performance is not linear, so it is difficult to know whether influencing one factor with the proposed intervention will result in measureable differences, especially in such a small group over such a short time period.

4. Approach:

Strengths

- The project is in its early stages so the scope of the proposal is relatively small scale. It is set up to answer a number of questions about intermittent claudication including clinical outcomes (walking distance) and biochemical parameters. I think there is a nice combination of clinical and physiologic questions.

Weaknesses

- Most claudication studies are carried out for at least three months, and the investigators have previously done three month studies. The rationale for making this a two month study is not explained. It is questionable whether a two month study with such a small patient cohort (12 patients each group) will be adequate to see a difference in the test parameters.
- Muscle biopsy is an invasive procedure in patients with limb ischemia. While the authors have extensive experience with this, I question how accepting patients will be of this procedure. Perhaps a little more description of how this is done would be helpful. For example, many patients with intermittent claudication are taking antiplatelet agents such as aspirin and clopidogrel. How will medications be managed around the time of the biopsy?
- It is not specifically stated how patients will be recruited except to say that they will be recruited from Durham NC and the surrounding communities. More detail on how patients will be recruited would be helpful.

5. Environment:

Strengths

- Scientific work environment appears strong with three separate facilities (Duke, Wake Forest, Virginia) involved utilizing different areas of expertise at the three facilities. There seems to be a good collaborative environment shared by the groups involved.
- Institutional support, equipment and physical resources appear adequate for the proposed research.

Weaknesses

- No specific weaknesses

Protections for Human Subjects:

- Protection of human subjects adequately addressed. There appears to be minimal potential harm to recruited patients. One potential area of harm is the muscle biopsy, although the investigators report extensive experience with this procedure.

Data and Safety Monitoring Plan (Applicable for Clinical Trials Only):

Inclusion of Women, Minorities and Children:

- Inclusion of women and minorities is addressed. It is stated that the targeted enrollment is 50% women and 50% minorities, but the specifics of how this targeted enrollment is to be achieved are not addressed
-

Vertebrate Animals:

- Not applicable

Biohazards:

- Not applicable

Resubmission:

- Not applicable

Renewal:

- Not applicable

Revision:

- Not applicable

Applications from Foreign Organizations:

- Not applicable

Resource Sharing Plans:

- Not applicable

Budget and Period of Support:

Recommended as Requested:

- Budget seems appropriate for the scope of the work

CRITIQUE 2:

Significance: 2
Investigator(s): 1
Innovation: 2
Approach: 2
Environment: 1

Overall Impact: This pilot study will test an oral high nitrate supplement that raises plasma nitrite in addition to 8 weeks of supervised exercise training in patients with claudication. The hypothesis is that the plasma nitrite may be transported to peripheral tissue and converted to nitric oxide under hypoxic

conditions. Exercise training in PAD induces ischemia repeatedly during each session, thus underscoring the potential of such an agent in this disease state. Thus those consuming the nitrate supplement could have greater improvement in pain claudication onset time and peak walking time than those undergoing exercise training alone. They also intend to evaluate the changes in tissue oxygenation during exercise between groups via NIRS and changes in gastrocnemius muscle tissue obtained through skeletal muscle biopsy. The protocol is very well designed and presented. The investigative team has the collective expertise to successfully complete the study and if effective, could add an additional treatment option for patients with claudication, for whom therapeutic options are limited.

1. Significance:

Strengths

- Oral agents that are effective in claudication have been very elusive despite considerable effort to develop such agents. Should this supplement prove to be effective, it would be very significant for patients with claudication.

Weaknesses

Weaknesses

- Minimal

2. Investigator(s):

Strengths

- This is a strong investigative team with the collective expertise recruit the subjects, perform the measurements proposed, deliver the interventions, and analyze the data.

Weaknesses

- None identified

3. Innovation:

Strengths

- The mechanism of this supplement triggered by the ischemic state seen with exertion in PAD is novel
- Examining changes in tissue oxygenation in the dynamic state while exercising as well as changes in skeletal muscle tissue in response to the intervention is also novel.

Weaknesses

- Treadmill exercise training is not novel, but appropriate for this protocol.

4. Approach:

Strengths

- This study is well designed and described in the application.
- Procedures are clear
- Preliminary data are provided

- Potential problems are addressed

Weaknesses

- Minimal

5. Environment:

Strengths

- The environment has the resources required to successfully conduct the protocol

Weaknesses

- None identified

Protections for Human Subjects:

Acceptable Risks and/or Adequate Protections

- The procedures that will be followed should an adverse event occur could be added to strengthen the application

Data and Safety Monitoring Plan (Applicable for Clinical Trials Only):

Not Applicable (No Clinical Trials)

Inclusion of Women, Minorities and Children:

G1A - Both Genders, Acceptable

M1A - Minority and Non-minority, Acceptable

C3A - No Children Included, Acceptable

- No concerns

Vertebrate Animals:

Not Applicable (No Vertebrate Animals)

Biohazards:

Not Applicable (No Biohazards)

Budget and Period of Support:

Recommend as Requested

CRITIQUE 3:

Significance: 1
Investigator(s): 2
Innovation: 1
Approach: 3
Environment: 2

Overall Impact: Peripheral artery disease (PAD) is common. Other than supervised exercise, there is a paucity of useful treatments for intermittent claudication. The present study, supported by animal data and by preliminary human data, suggests that dietary nitrates may improve exercise performance in PAD, by raising blood nitrite levels which may serve as a source to nitric oxide at sites of ischemia.

The applicants wish to conduct a study to investigate dietary nitrates for the treatment PAD/intermittent claudication. In a pilot placebo controlled parallel design study, they will look for changes in exercise performance, nitric oxide bioavailability and for evidence of enhanced angiogenesis in skeletal muscle biopsies.

This study is innovative and the pilot data generated by this R21 grant would serve to prepare a more definitive clinical trial. The investigators are expert in the proposed measurements and in a good position to complete the proposed aims. The study is significant as new treatments for intermittent claudication are much needed.

1. Significance:

2. Investigator(s):

3. Innovation:

4. Approach:

Strengths

- Careful attention is paid to most experimental details. Collaborations are in place, including the analysis of muscle biopsies, to accomplish the aims.

Weaknesses

- Why assume that improvements in exercise with nitrate/nitrite are due to more efficient distribution of blood flow and not due to altered skeletal muscle and/or mitochondrial bioenergetics related to increases in nitric oxide (e.g. Journal of Cell Science 119, 2855-2862)? This single focus on local blood flow to the exclusion of skeletal muscle and mitochondrial function is one potential weakness in this grant.
- Given the variability of stress testing in PAD, is this rather small study adequate to inform about sample sizes for a larger study?
- The use of plethysmography to measure lower extremity blood flow is noted in the grant application. Will plethysmographic blood flow be assessed during reactive hyperemia (it is not stated)? The peak and particularly the duration of reactive hyperemia reflect NO bioavailability (e.g. Am J Physiol 1996; 270:H1435). This ischemic stimulus seems particularly relevant as it would create the conditions (i.e. ischemia) that facilitate conversion of nitrite to NO.

5. Environment:

Protections for Human Subjects:

Acceptable Risks and/or Adequate Protections

Data and Safety Monitoring Plan (Applicable for Clinical Trials Only):

Inclusion of Women, Minorities and Children:

G1A - Both Genders, Acceptable

M1A - Minority and Non-minority, Acceptable

C3A - No Children Included, Acceptable

Vertebrate Animals:

Not Applicable (No Vertebrate Animals)

Biohazards:

Not Applicable (No Biohazards)

Budget and Period of Support:

Recommended budget modifications or possible overlap identified:

- Dr. Gary Miller's request for 3.0 month effort is not well justified. 1.0 month seems more appropriate to the stated contribution.

THE FOLLOWING RESUME SECTIONS WERE PREPARED BY THE SCIENTIFIC REVIEW OFFICER TO SUMMARIZE THE OUTCOME OF DISCUSSIONS OF THE REVIEW COMMITTEE ON THE FOLLOWING ISSUES:

PROTECTION OF HUMAN SUBJECTS (Resume): ACCEPTABLE

INCLUSION OF WOMEN PLAN (Resume): ACCEPTABLE

INCLUSION OF MINORITIES PLAN (Resume): ACCEPTABLE

INCLUSION OF CHILDREN PLAN (Resume): ACCEPTABLE

COMMITTEE BUDGET RECOMMENDATIONS: The budget was recommended as requested.

+ Derived from the range of percentile values calculated for the study section that reviewed this application.

NIH has modified its policy regarding the receipt of resubmissions (amended applications). See Guide Notice NOT-OD-10-080 at http://grants.nih.gov/grants/guide/notice-files/NOT-OD-10-080.html.
The impact/priority score is calculated after discussion of an application by averaging the overall scores (1-9) given by all voting reviewers on the committee and multiplying by 10. The

criterion scores are submitted prior to the meeting by the individual reviewers assigned to an application, and are not discussed specifically at the review meeting or calculated into the overall impact score. For details on the review process, see http://grants.nih.gov/grants/peer_review_process.htm#scoring.

MEETING ROSTER

Clinical and Integrative Cardiovascular Sciences Study Section
Cardiovascular and Respiratory Sciences Integrated Review Group
CENTER FOR SCIENTIFIC REVIEW
CICS
June 16, 2011 - June 17, 2011

ACTING CHAIR

KRAMER, CHRISTOPHER M, MD
PROFESSOR
DIRECTOR, CARDIOVASCULAR IMAGING
UNIVERSITY OF VIRGINIA HEALTH SYSTEM
CHARLOTTESVILLE, VA 22908

MEMBERS

BABB, JOSEPH D, MD *
PROFESSOR
DEPARTMENT OF INTERNAL MEDICINE
EAST CAROLINA HEART INSTITUTE
BRODY SCHOOL OF MEDICINE
EAST CAROLINA UNIVERSITY
EAST CAROLINA UNIVERSITY
GREENVILLE, NC 27835

BASNAKIAN, ALEXEI G, MD, PHD *
ASSOCIATE PROFESSOR
DEPARTMENT OF PHARMACOLOGY
AND TOXICOLOGY
UNIVERSITY OF ARKANSAS FOR MEDICAL SCIENCES
LITTLE ROCK, AR 72205

BOEHMER, JOHN P, MD
PROFESSOR
DEPARTMENTS OF MEDICINE AND SURGERY
DIVISION OF CARDIOLOGY
THE MILTON S. HERSHEY MEDICAL CENTER
PENNSYLVANIA STATE UNIVERSITY
HERSHEY, PA 17033

CALHOUN, DAVID A, MD
PROFESSOR OF MEDICINE
VASCULAR BIOLOGY AND HYPERTENSION PROGRAM
DIVISION OF CARDIOVASCULAR DISEASE
UNIVERSITY OF ALABAMA AT BIRMINGHAM
BIRMINGHAM, AL 35294

CARTER, JASON R, PHD *
CHAIR & ASSOCIATE PROFESSOR
DEPARTMENT OF KINESIOLOGY & INTEGRATIVE
PHYSIOLOGY
MICHIGAN TECHNOLOGICAL UNIVERSITY
HOUGHTON, MI 49931

CAVALLARI, LARISA HUMMA, PHMD *
ASSISTANT PROFESSOR
DEPARTMENT OF PHARMACY PRACTICE
UNIVERSITY OF ILLINOIS AT CHICAGO
CHICAGO, IL 60612

DIAZ, FRANCISCO , PHD *
ASSOCIATE PROFESSOR
DEPARTMENT OF BIOSTATISTICS
THE UNIVERSITY OF KANSAS MEDICAL CENTER
KANSAS CITY, KS 66160

DIVINE, GEORGE W, PHD *
SENIOR RESEARCH BIOSTATISTICIAN
DEPARTMENT OF BIOSTATISTICS
AND RESEARCH EPIDEMIOLOGY
HENRY FORD HEALTH SYSTEM
DETROIT, MI 48202

FEINSTEIN, JEFFREY A., MD *
ASSOCIATE PROFESSOR
DEPARTMENT OF PEDIATRICS-CARDIOLOGY
STANFORD UNIVERSITY SCHOOL OF MEDICINE
STANFORD, CA 94305

GAGNON, DAVID , MD, PHD *
ASSOCIATE PROFESSOR
DEPARTMENT OF EPIDEMIOLOGY & BIOSTATISTICS
BOSTON UNIVERSITY SCHOOL PF PUBLIC HEALTH
BOSTON, MA 021182526

GANZ, PETER , MD
MAURICE ELIASER, JR, M.D. DISTINGUISHED
PROFESSOR
CHIEF, DIVISION OF CARDIOLOGY
DIRECTOR, CENTER OF EXCELLENCE IN VASCULAR
RESEARC
SAN FRANCISCO GENERAL HOSPITAL
UNIVERSITY OF CALIFORNIA, SAN FRANCISCO
SAN FRANCISCO, CA 94110

GAZMURI, RAUL JAIME, MD, PHD
PROFESSOR
SECTION CHIEF, CRITICAL CARE MEDICINE
ROSALIND FRANKLIN UNIVERSITY
OF MEDICINE AND SCIENCE
NORTH CHICAGO, IL 60064

HOELSCHER, THILO MD, MD *
ASSISTANT PROFESSOR
DEPARTMENT OF RADIOLOGY & NEUROSCIENCES
DIRECTOR, BRAIN ULTRASOUND RESEARCH
LABORATORY
UNIVERSITY OF CALIFORNIA, SAN DIEGO
SAN DIEGO, CA 921038756

KITCHEN, CHRISTINA MICHELLE RAMIREZ, PHD
ASSOCIATE PROFESSOR
DEPARTMENT OF BIOSTATISTICS
SCHOOL OF PUBLIC HEALTH
UNIVERSITY OF CALIFORNIA AT LOS ANGELES
LOS ANGELES, CA 90095

KLEINDORFER, DAWN O, MD *
PROFESSOR
VASCULAR NEUROLOGY DIVISION DIRECTOR
UNIVERSITY OF CINCINNATI COLLEGE OF MEDICINE
CINCINNATI, OH 452670525

LANDRY, GREGORY J, MD *
ASSOCIATE PROFESSOR
VASCULAR SURGERY
DEPARTMENT OF SURGERY, SCHOOL OF MEDICINE
OREGON HEALTH & SCIENCE UNIVERSITY
PORTLAND, OR 97239

LE-RADEMACHER, JENNIFER G. *
ASSISTANT PROFESSOR
DIVISION OF BIOSTATISTICS
MEDICAL COLLEGE OF WISCONSIN
MILWAUKEE, WI 53226

MAJOR, AMY S, PHD *
ASSISTANT PROFESSOR
DEPARTMENT OF MEDICINE
DIVISION OF CARDIOVASCULAR MEDICINE
VANDERBILT UNIVERSITY
NASHVILLE, TN 37232

MEGGS, LEONARD GERALD, MD *
PROFESSOR & CHAIRMAN
DEPARTMENT OF NEPHROLOGY
OCHSNER CLINICAL FOUNDATION
NEW ORLEANS, LA 70121

NEWTON-CHEH, CHRISTOPHER HOLMES, MD *
ASSISTANT PROFESSOR
CENTER FOR HUMAN GENETIC RESEARCH
CARDIOVASCULAR RESEARCH CENTER
MASSACHUSETTS GENERAL HOSPITAL
BOSTON, MA 02114

PINA, ILEANA L MDMPH, MD *
PROFESSOR OF MEDICINE
SECTION OF HEART FAILURE AND TRANSPLANTATION
DIVISION OF CARDIOLOGY
CASE WESTERN RESERVE UNIVERSITY
CLEVELAND, OH 44106

SCROGIN, KARIE E, PHD
ASSOCIATE PROFESSOR
DEPARTMENT OF PHARMACOLOGY
STRITCH SCHOOL OF MEDICINE
LOYOLA UNIVERSITY
MAYWOOD, IL 60153

SWEITZER, NANCY K, MD, PHD *
ASSOCIATE PROFESSOR
DIRECTOR, HEART FAILURE PROGRAM
DEPARTMENT OF MEDICINE
UNIVERSITY OF WISCONSIN
MADISON , WI 53792

TREAT-JACOBSON, DIANE J, PHD
ASSOCIATE PROFESSOR
SCHOOL OF NURSING
UNIVERSITY OF MINNESOTA
MINNEAPOLIS, MN 55455

URBINA, ELAINE M, MD
ASSOCIATE PROFESSOR
DIRECTOR OF PREVENTIVE CARDIOLOGY
CINCINNATI CHILDREN'S HOSPITAL MEDICAL CENTER
CINCINNATI, OH 45229

VALDERRABANO, MIGUEL , MD *
CHIEF, DIVISION OF CARDIAC ELECTROPHYSIOLOGY
METHODIST DEBAKEY HEART AND VASCULAR CENTER
HOUSTON, TX 77030

WEBB, NANCY R, PHD
PROFESSOR
DEPARTMENT OF INTERNAL MEDICINE
CARDIOVASCULAR RESEARCH CENTER
UNIVERSITY OF KENTUCKY
LEXINGTON, KY 40536

WOODARD, PAMELA K, MD
PROFESSOR
CARDIOVASCULAR IMAGING LABORATORY
MALLINCKRODT INSTITUTE OF RADIOLOGY
WASHINGTON UNIVERSITY SCHOOL OF MEDICINE
ST. LOUIS, MO 63110

YAU, C LILLIAN, PHD *
PRIVATE PRACTICE
STERLING, VA 20165

MAIL REVIEWER(S)

CURTIS, ANNE B, MD
PROFESSOR
PROFESSOR AND CHAIR
DEPARTMENT OF MEDICINE
UNIVERSITY OF BUFFALO
BUFFALO, NY 14215

JONAS, RICHARD A, MD
PROFESSOR
DIVISION CHIEF, CARDIAC SURGERY
CHILDREN'S NATIONAL HEART INSTITUTE
WASHINGTON, DC 20010

MAGALANG, ULYSSES J MD, MD
PROFESSOR
DIVISION OF PULMONARY, ALLERGY, CRITICAL CARE &
SLEEP MEDICINE
THE OHIO STATE UNIVERSITY
COLUMBUS, OH 43210

RAJAGOPALAN, SANJAY , MD, FACC
WOLFE PROFESSOR OF MEDICINE & RADIOLOGY
DIRECTOR, VASCULAR MEDICINE &
CO-DIRECTOR MR/CT IMAGING
THE OHIO STATE UNIVERSITY
COLUMBUS, OH 43210

SUNDEL, ROBERT PICARD
ASSOCIATE PROFESSOR
DIVISION OF IMMUNOLOGY
CHILDREN'S HOSPITAL BOSTON
BOSTON, MA 021155737

SCIENTIFIC REVIEW ADMINISTRATOR

DOWELL, RUSSELL T, PHD
SCIENTIFIC REVIEW OFFICER
CENTER FOR SCIENTIFIC REVIEW
NATIONAL INSTITUTES OF HEALTH
BETHESDA, MD 20892

GRANTS TECHNICAL ASSISTANT

FERGUSON, JAMES A
GRANTS TECHNICAL ASSISTANT
CENTER FOR SCIENTIFIC REVIEW
NATIONAL INSTITUTES OF HEALTH
BETHESDA, MD 20892

* Temporary Member. For grant applications, temporary members may participate in the entire meeting or may review only selected applications as needed.

Consultants are required to absent themselves from the room during the review of any application if their presence would constitute or appear to constitute a conflict of interest.
